# Supplementary material for: Identification of photoperiod-induced specific miRNAs in the adrenal glands of Sunite sheep (Ovis aries)
Source: Front Vet Sci. 2022 Jul 22;9:888207. doi: 10.3389/fvets.2022.888207 (PMC9354845; doi:10.3389/fvets.2022.888207)
Supplement: Supplementary file 1 [file Data_Sheet_1.ZIP › Supplementary file/miRDeep2 file.zip!/miRDeep2 file/Novel_Structure/LP42A1/Novel_255.pdf]

Provisional ID	:	NC_019468.2_19978
Score total	:	1.5
Score for star read(s)	:	-1.3
Score for read counts	:	0
Score for mfe	:	1.2
Score for randfold	:	1.6
Score for cons. seed	:	
Total read count	:	113
Mature read count	:	113
Loop read count	:	0
Star read count	:	0

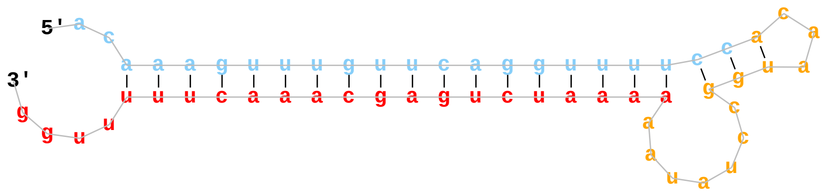

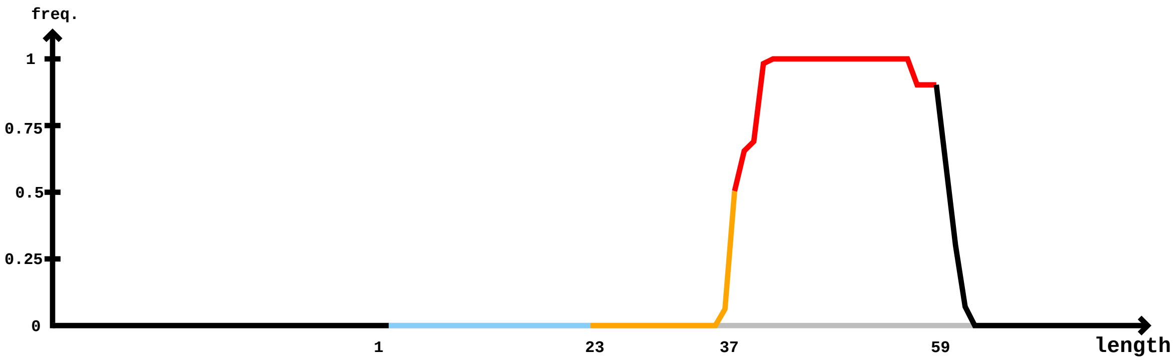

Star Mature

```
5'- ggagaucgagagcagaaaaauacugggcuggccacaaaguuuguucagguuuuccacaauggccuauaaaaaaucugagcaaacuuuuuggccaacacaauaaaaggaccag   -3'    exp
    ......................((((..((((((.(((((((((((((((((((((...))).......)))))))))))))))))).)))))).............. ))))    reads    mm    sample
    ....................................................................aaaaaCcugagcaaacuuuu........................    7        1     seq
    .....................................................................aaaaucugaAcaaacuuuu........................    4        1     seq
    .....................................................................aaaaucugaAcaaacuuuuugg.....................    45       1     seq
    .....................................................................aaaaucugaAcaaacuuuuuggc....................    1        1     seq
    ......................................................................aaaucugaAcaaacuuuuugg.....................    14       1     seq
    ......................................................................aaaucugagcaaacuuuuuggc....................    3        0     seq
    .......................................................................aaucugaAcaaacuuuuuggc....................    4        1     seq
    ........................................................................aucugaAcaaacuuuuuggcc...................    25       1     seq
    ........................................................................aucugaAcaaacuuuuuggcca..................    8        1     seq
    .........................................................................ucugaAcaaacuuuuuggc....................    1        1     seq
    .........................................................................ucugagcaaacuuuuuggcc...................    1        0     seq
```
